# Fertility preservation in gynecologic cancer patients: Number 3 – March 2023

**DOI:** 10.1055/s-0043-1768564

**Published:** 2023-05-24

**Authors:** Suzana Arenhart Pessini, Jesus Paula Carvalho, Ricardo dos Reis, Agnaldo Lopes da Silva Filho, Walquíria Quida Salles Pereira Primo

**Affiliations:** 1 Universidade Federal do Rio Grande do Sul, Porto Alegre, RS, Brazil; 2 Universidade de São Paulo, São Paulo, SP, Brazil; 3 Hospital de Câncer de Barretos, Barretos, SP, Brazil; 4 Universidade Federal de Minas Gerais, Belo Horizonte, MG, Brazil; 5 Universidade de Brasília, Brasília, DF, Brazil

## Keypoints

- Fertility preservation in cancer patients is possible through conservative surgery or assisted reproduction techniques.
- Patients of childbearing age with a diagnosis of malignancy benefit from reproductive counseling.
- Oncological and reproductive information results in lower rates of regret, even when the patient chooses to abandon conservative treatment.
- Criteria for indicating conservative treatment in cervical, endometrial and ovarian cancer are presented in this position statement.
- Conservative treatment modalities in cervical, endometrial and ovarian cancer are discussed together with the respective oncological and reproductive outcomes.

## Recommendations

- Inform the patient about the types of conservative treatment, oncological outcomes and reproductive chances.
- Patients with histological diagnosis of cervical and endometrial cancer should be properly staged by clinical examination and imaging tests. Magnetic resonance imaging (MRI) is the imaging method that best defines tumor size and preoperative extent of the neoplasm.
- In patients with early cervical cancer with squamous, adenocarcinoma, and adenosquamous histologies, possible conservative surgeries are: conization with margins free of tumor and free of high-grade squamous intraepithelial lesion (HSIL) at stage IA1 without lymphovascular space involvement (LVSI). Radical trachelectomy with pelvic lymphadenectomy at stages IA1 with LVSI, IA2 and IB1 (up to 2 cm) with or without LVSI. There is no indication of radical treatment after pregnancy.
- Other fertility-sparing surgeries on the cervix include oophoropexy and uterine transposition.
- Patients with stage IA well-differentiated endometrioid histologic type endometrial cancer may be treated with a levonorgestrel-releasing intrauterine system (LNG-IUS) and/or high-dose progestins, either orally or intramuscularly. Hysteroscopic tumor resection preceding hormone therapy has better results. After conservative treatment of endometrial cancer, it is recommended that pregnancy occurs as soon as there is neoplasm remission.
- Suspected ovarian cyst in a patient with reproductive desire should be evaluated by ultrasound performed by an experienced professional associated with tumor markers.
- Fertility-sparing surgery in ovarian cancer consists of preserving the uterus with or without preservation of the contralateral annex. Young patients with low-grade stage IA epithelial histology (G1 and G2), stage IA/IC non-epithelial histology and low malignant potential (borderline) are candidates. Complementation of surgery is recommended after the end of pregnancy for patients with invasive epithelial disease, and is not necessary for non-epithelial and borderline tumors.
- Assisted reproduction techniques, such as cryopreservation of oocyte, embryo or ovarian tissue may be necessary and thus offered to patients.

## Background

Gynecological cancer directly affects fertility, as treatment consists of surgical removal of the reproductive system and/or exposure to gonadotoxic agents. However, patients in early stages who meet established criteria can be treated with fertility-sparing surgeries and reach equivalent oncological results to those of traditional treatments. Fertility preservation techniques such as cryopreservation of oocytes, embryos and ovarian tissue may also be offered in some situations. The American Society of Clinical Oncology (ASCO) has published recommendations on fertility preservation with the aim of raising awareness on the topic, and, together with the American Society for Reproductive Medicine (ASRM), they recommend that patients of childbearing age with cancer undergo reproductive counseling. These patients have lower rates of regret, even when they choose to abandon conservative treatment. 1 Interest in fertility preservation has increased in recent decades, both because women delay pregnancy and because of the higher incidence of cancer in young people. The incidence rate of all cancers increased by 29% between 1973 and 2015 in adolescents and young adults of both sexes. 2 Cervical cancer in women aged 20-29 years increased annually by an average of 10.3% between 2000 and 2009. 3 Failure to advise cancer patients about the possibilities of preserving fertility may raise questions in the future; in some countries, this is already considered medical malpractice.

## Cervical cancer

Because it affects young women, has high incidence and mortality rates, cervical cancer has great importance among gynecological tumors. In the United States of America, about half of all fertile women diagnosed with early-stage cervical cancer meet the criteria for conservative surgery. Once the histological diagnosis is made, staging is the initial condition for treatment.

### When are conservative surgeries indicated?

**Conization:** In stage IA1 without LVSI, conization or trachelectomy with surgical margins free of tumor and free of HSIL serves as diagnosis and treatment in women who wish to preserve the uterus. It should be a single non-fragmented specimen. 4 5 6

**Radical trachelectomy with pelvic lymphadenectomy:** The preferred treatment in stages IA1 with LVSI, IA2 and IB1 with or without LVSI. 4 5 6 7 The surgery is performed vaginally, associated with laparoscopic pelvic lymph node dissection, laparotomy abdominal approach or minimally invasive surgery (MIS), videolaparoscopic or robotically. In the International Radical Trachelectomy Assessment (IRTA) study, open surgery was compared with minimally invasive surgery, and no difference in survival and recurrence was found, although further studies are needed to confirm the safety of MIS. 8

The criteria to be followed are reproductive age, desire to preserve fertility, tumors of up to 2 cm (the greatest diameter), squamous, adenocarcinoma and adenosquamous histological types, absence of parametrial invasion, lymph node metastasis and infertility. Other histological types, such as neuroendocrine and non-human papillomavirus (HPV)-associated adenocarcinoma are contraindicated for conservative treatment. 4 5 6 7 Magnetic resonance imaging (MRI) is the best imaging method to assess the preoperative extent of the neoplasm, such as tumor size, depth of stromal invasion, distance between the upper part of the tumor and the internal orifice, lymph node metastasis, and parametrial invasion. 4 5 6 Positron emission tomography (PET) is superior to MRI and computed tomography (CT) in the evaluation of lymph node metastasis.

### Sentinel lymph node

The the sentinel lymph node mapping and immunohistochemical analysis is recommended for the identification of low-volume metastases (isolated tumor cells and micrometastases). The anatomopathological analysis of intraoperative frozen section has the advantage of contraindicating surgery, in addition to allowing ovarian transposition in the same surgical time. The disadvantage is the risk of not identifying low-volume metastases. 9 The International Federation of Gynecology and Obstetrics (FIGO) suggests freezing sentinel lymph nodes and, if negative, completing the surgery or, alternatively, trachelectomy in a second time after the anatomopathological paraffin examination of the lymphadenectomy. 4

### What is the role of parametrectomy?

The need for parametrectomy in low-risk stages IA2 and IB1 is the subject of study. In a meta-analysis comparing simple trachelectomy or conization with radical trachelectomy, similar oncological results were found, with less fetal loss in the conization groups. 10 The prospective ConCerv study comprised the analysis of simple hysterectomy or conization + pelvic lymphadenectomy in stage IB1 patients in specimens of conization with free margins, without LVSI and without suspected lymph node metastasis, squamous and adenocarcinoma histological types, and the recurrence rate was similar to that of radical treatment. 11 After the completion of two other ongoing studies – SHAPE and GOG 278 – that aim to compare the oncological results of simple hysterectomy and radical hysterectomy in early stages, the evidence will be more robust regarding the need for parametrectomy.

### Tumors larger than 2 cm

In patients with tumors larger than 2 cm, neoadjuvant chemotherapy is a possibility. The cisplatin paclitaxel regimen is the most commonly used, even though carboplatin and paclitaxel have less toxicity. A recent meta-analysis showed 39% of pathological complete response and 45.6% of partial response. 12 The multicenter CONTESSA study, scheduled to end in 2025, estimates a good response in more than 70% of patients. It remains unclear if lymph node dissection should be performed before chemotherapy, if the surgery to be performed after chemotherapy is radical trachelectomy or conization, and what is the best chemotherapy regimen.

### Oncology results

Conservative surgery performed according to indication criteria does not differ from radical hysterectomy in terms of oncological safety. 4 5 6 The recurrence and 5-year mortality rates of radical trachelectomy are 3-6% and 1.6-5%, respectively. 13 14 Two systematic reviews analyzed different approaches to radical trachelectomy. Recurrence and death from cancer were, respectively, 4% and 1.7-2% vaginally, 4.7% and 1.4% in laparotomy, and 7.5% and 1.3% in the laparoscopic route. Recurrence rates were associated with tumor size greater than 2 cm and LVSI. 13 14

### Reproductive results

Infertility after radical trachelectomy occurs in 14-41%, and some patients may require assisted reproduction techniques. 15 Although first trimester abortion is comparable to that of the general population, second trimester miscarriage is more frequent. Prematurity occurs in 28-38% of pregnant women and, before 32 weeks in 12%. 14 16 Fetal loss in the second trimester and prematurity before 32 weeks result from premature rupture of membranes secondary to cervical insufficiency. Cerclage can be performed vaginally, but the abdominal route has better results. It is preferably performed at the same surgical time. Pregnancy rates range from 55-65.8% and the rate of live newborns is 70%. 14 17 In the series by Speiser et al., of the 212 patients treated, 76 (35.8%) were planning to become pregnant up to five years after surgery. Fifty out of these 76 became pregnant, resulting in a pregnancy rate of 65.8%. However, the pregnancy rate for all 212 patients was 24% (50/212). 17 Pregnancy is considered high risk and antenatal care is performed at a referral center. As for specific procedures to be adopted for these patients, evidence is scarce and based only on observational studies. Vaginal progesterone and cerclage, investigation of asymptomatic bacteriuria, and cervical length follow-up by ultrasound are suggested. Elective cesarean section is preferred. 9

### How is the follow-up performed?

Reviews every 3-4 months in the first two years, every six months from the third to the fifth year, and annually thereafter. 4 5 6 In addition to anamnesis and physical examination, cervicovaginal cytology is recommended annually. A follow-up period of 6-12 months is advised for pregnancy. 1 There is no indication of radical treatment after pregnancy.

### What are the alternatives for conservative surgery?

Ovarian transposition or oophoropexy conserves ovarian function by suspending the gonads out of the radiation field. The ovaries are fixed above the iliac crests and clips are placed to guide the radiotherapist. The dose of pelvic radiotherapy for cervical cancer is 40 to 50 Gy, and ovarian failure occurs at lower doses, between 2 and 12 Gy. The best results are in patients younger than 40 years and after brachytherapy, compared with external beam radiotherapy. When cryopreservation is intended, oocyte aspiration is preferably performed during surgery. Note that ovarian transposition is justified mainly for the maintenance of fertility and oocyte capture, and less justified for the maintenance of hormonal function. Another feasible surgery is uterine transposition, initially proposed for cancer of the rectum and other pelvic tumors that require irradiation. In 2020, it was described in a patient with cervical cancer undergoing fertility-sparing surgery who required external radiotherapy due to micrometastasis in pelvic lymph nodes. 18

## Endometrial cancer

It mainly affects postmenopausal women, even though 4% occur before the age of 40 years and 6.4% between 20 and 44 years of age. 19 In these age groups, tumors are generally well differentiated. Around 10% are associated with Lynch Syndrome (LS).

### How is conservative treatment performed and when is it possible?

The preservation of the uterine body, fallopian tubes and ovaries in patients with endometrial carcinoma is limited to the well-differentiated endometrioid histological type (G1), stage IA without myometrial infiltration. In addition to clinical and family history, with attention to the possibility of LS, the patient is advised about weight loss and informed about the risks and the need to complement treatment after pregnancy. Referral to a bariatric surgeon may be necessary and if there are other comorbidities, to a specialist in preconception counseling. 1 Magnetic resonance is the imaging method that best defines myometrial invasion, cervical invasion, and lymph node metastasis. 20 21 The preferred conservative treatment is hormonal with oral systemic progestogen, such as medroxyprogesterone acetate (MPA) or megestrol acetate (MA), or with a intrauterine levonorgestrel device (LNG-IUD). Medroxyprogesterone acetate doses range from 2.5 to 1,500 mg/day, more frequently between 400-600 mg/day. 20 21 The reported doses of MA are from 10 to 400 mg/day, most commonly 160-320 mg/day. 20 22 Evidence is limited on which one is more effective, what is the duration of treatment, and what is the safest dose. Some studies suggest better responses with lower doses, such as 10 mg/day of MPA and 160 mg/day of MA. 22 23 Treatment duration varies from eight weeks to nine months. Side effects may occur, such as weight gain, thrombosis, mood swings, headache and breast tension. LNG-IUD can be used alone or in combination with systemic progestogen, with the combination considered preferred. 1 24 Hysteroscopic resection of the tumor and adjacent endometrium preceding LNG-IUD or progestogen has better rates of complete response, higher rates of pregnancy and fewer hysterectomies. 25 Other medications are proposed, such as GnRH analogues, aromatase inhibitors and metformin. The risk of recurrence or persistent disease is greater with conservative treatment compared to hysterectomy, and surgical staging is indicated after pregnancy. Even at a presumed early stage, the risk of synchronous ovarian cancer is 4-25% in women younger than 45 years. 26 Another warning factor is the possibility that LS may be involved in the etiology of the tumor in a young patient whose molecular diagnosis is difficult with conservative treatment. An alternative to conservative surgery is preservation of only the macroscopically normal ovaries. In a study of women with G1 endometrial carcinoma under 50 years of age who underwent surgery, survival was significantly higher in the group that had their ovaries preserved given the lower cumulative risk of cardiovascular disease. 27

### Oncology results

Complete response rates range from 48% to 96%, considering all types of treatment. 24 28 Recurrence among patients who achieve complete response ranges from 25% to 47%. 1 22 24 In terms of efficacy, oral progestogens have more side effects and greater recurrence than LNG-IUD, although data are still inconsistent. 21 Treatment with LNG-IUD with or without oral progestin results in a complete response in 63-96% of patients. 24 29 30 In a recent meta-analysis, complete response occurred in 79.7%, with 35.3% of recurrence. 31 In a randomized study of patients with G1 adenocarcinoma and atypical hyperplasia treated with LNG-IUD alone compared to the combination of weight loss, and metformin use, complete response rates after six months were 61%, 67%, and 57%, respectively. Considering adenocarcinoma and atypical hyperplasia in the three groups, remission occurred in 43% and 82%, respectively. 32

### Obstetric results

Pregnancy rates vary from 32-53% and rates of live births from 28-69.4%. 22 31 The highest chances occur in women up to 35 years of age, with treatment combining hysteroscopic resection + progestogens and with up to three years of follow-up. 31 In the series treated with hysteroscopic resection and LNG-IUD, the rate of live births was 83% among the 63% of patients who achieved a complete response. 28 Pregnancy is recommended as soon as neoplasm remission is achieved (two negative biopsies), since there is a risk of recurrence.

### How should patient follow-up be?

The patient is alerted about bleeding and given lifestyle advice. The first histological control is performed three months after the start of treatment. In the case of complete response, histological control is quarterly. Pregnancy is suggested after two negative endometrial samples. 1 If there is no response, increase the progestin dose and follow the quarterly control. If there is no response or progression after nine months, definitive surgical treatment is indicated. 21

## Ovarian cancer

Ovarian cancer is less common than cervical and uterine cancer, but it is the most lethal. Most occur after menopause and 11.8% occur before the age of 45 years, generally at an early stage and with a better prognosis. 19 33

### How to manage the patient with a desire to become pregnant?

The evaluation of the patient with a pelvic mass includes, in addition to anamnesis and physical examination, an ultrasound performed by an experienced professional. A family history of ovarian cancer is the most important risk factor to consider. Although tumor markers help, they are unspecific; CA-125, alpha-fetoprotein (α-FP), human chorionic gonadotropin (hCG) and lactate dehydrogenase (LDH) are the most used. The most frequent histological types in childhood and adolescence are germ cell types and, in reproductive adulthood, epithelial types. The patient should be informed that intraoperative frozen section has limitations, with sensitivity and specificity around 90% and 99.5%, respectively. When the frozen section diagnosis is a borderline tumor, in 21% of the cases the result in the paraffin will be an invasive tumor. 34 Therefore, the fertility-sparing surgical planning may change after the final histopathological result. For this reason, many authors suggest the management of suspicious ovarian lesions in two steps in patients who wished to maintain fertility, awaiting the definitive histopathology for decision making. Fertility-sparing surgery provides for preservation of the uterus with or without preservation of the contralateral annex. It is acceptable in young patients with low-grade stage IA epithelial histology (G1 and G2), non-epithelial germ cell and sex cord stromal histology stage IA/IC and low malignant potential (borderline). 35 36 Approximately one-third of borderline tumors occur in women under 40 years of age. In stage I, survival rates reach 99%, and unilateral salpingo-oophorectomy associated with collection of peritoneal lavage, omentectomy and biopsy of any peritoneal alteration is the conservative treatment option. 35 36 Considering that the definitive histologic diagnosis may change the therapeutic plan, oocyte or embryo cryopreservation is advised in patients with an ovarian tumor suspected of malignancy.

### Conservative surgery results

A systematic review of 120 studies resulted in 54% of pregnancy in patients treated conservatively for borderline tumors. 37 According to a recent study, there was no worse obstetric outcome in pregnancies after fertility-sparing surgery for ovarian cancer compared to low-risk pregnancies. 38 Complementation of surgery is recommended after termination of pregnancy for patients with invasive epithelial disease, and is not necessary for non-epithelial or borderline tumors.

### How should follow-up of the patient with conservative surgery be?

Follow-up is quarterly in the first two years and every six months between the third and fifth years. Imaging examination is recommended annually.

## The importance of fertility assessment and assisted reproduction techniques

Patients with cancer and the desire to preserve fertility, when evaluated by a reproduction specialist, have better conditions for a safe choice, as they receive information about age and fertility, ovarian reserve and their reproductive potential. The multidisciplinary discussion with clinical oncologist, radiotherapist, pathologist and psychologist is important for therapeutic planning and follow-up. In early stages, conservative surgeries are the first fertility-sparing options. However, it may be interesting or even necessary to add fertility preservation techniques that include oocyte, embryo or ovarian tissue cryopreservation. The first two are more widespread and in two weeks, ovarian hyperstimulation is performed. The main difference between these techniques is that the embryo belongs to the couple, while the oocyte belongs to the patient. More than half of patients with a partner prefer oocyte cryopreservation without fertilization or adhere to both techniques (oocyte and embryo cryopreservation). 39

## Final considerations

Fertility-sparing treatment in women with gynecological cancer is premised on the patient’s desire and potential to become pregnant, although without worsening the oncological outcome. Careful selection is one of the most critical phases of this process. The care of the patient candidate for conservative treatment must be multidisciplinary in a reference center, and reproductive counseling with a specialist in assisted reproduction is recommended. Sentinel lymph node and ultrastaging is stimulated in cervical-sparing surgery. The evaluation of the ovarian reserve and of reproductive possibilities is performed by a specialist in reproduction. Assisted reproduction techniques do not compromise the outcome and can add reproductive results to conservative surgical treatment.

National Commission Specialized in Gynecologic Oncology of the Brazilian Federation of Gynecology and Obstetrics Associations (Febrasgo)

President:

Walquíria Quida Salles Pereira Primo

Vice-president:

Suzana Arenhart Pessini

Secretary:

Jesus Paula Carvalho

Members:

Angélica Nogueira Rodrigues

Caetano da Silva Cardial

Delzio Salgado Bicalho

Eduardo Batista Candido

Etelvino de Souza Trindade

Fernando Maluf

Francisco José Cândido dos Reis

Georgia Fontes Cintra

Marcia Luiza Appel Binda

Mirian Helena Hoeschl Abreu Macedo

Renato Moretti Marques

Ricardo dos Reis

Sophie Françoise Mauricette Derchain

Heloisa de Andrade Carvalho

Filomena Marino Carvalho
